# Effects of Initial Water Content on Microstructure and Mechanical Properties of Lean Clay Soil Stabilized by Compound Calcium-Based Stabilizer

**DOI:** 10.3390/ma11101933

**Published:** 2018-10-10

**Authors:** Chenglong Yin, Wei Zhang, Xunli Jiang, Zhiyi Huang

**Affiliations:** College of Civil Engineering and Architecture, Zhejiang University, 866 Yuhangtang Road, Hangzhou 310058, China; yinchenglong@zju.edu.cn (C.Y.); zhangw8778@zju.edu.cn (W.Z.); 221712088@zju.edu.cn (X.J.)

**Keywords:** initial water content, clay soil, calcium-based stabilizer, soil stabilization, compressibility, strength, microstructure and composition

## Abstract

Initial water content significantly affects the efficiency of soil stabilization. In this study, the effects of initial water content on the compressibility, strength, microstructure, and composition of a lean clay soil stabilized by compound calcium-based stabilizer were investigated by static compaction test, unconfined compression test, optical microscope observations, environment scanning electron microscopy, energy dispersive X-ray spectroscopy, and X-ray diffraction. The results indicate that as the initial water content increases in the range studied, both the compaction energy and the maximum compaction force decrease linearly and there are less soil aggregates or agglomerations, and a smaller proportion of large pores in the compacted mixture structure. In addition, for specimens cured with or without external water supply and under different compaction degrees, the variation law of the unconfined compressive strength with initial water content is different and the highest strength value is obtained at various initial water contents. With the increase of initial water content, the percentage of the oxygen element tends to increase in the reaction products of the calcium-based stabilizer, whereas the primary mineral composition of the soil-stabilizer mixture did not change notably.

## 1. Introduction

From a historical perspective, clayey soil, derived from the rock weathering process of the geological cycle [1,2], already existed long before humankind. From the moment this material was used, its volume and strength changing property with seasonal moisture variation has frequently been detrimental to buildings and structures constructed on it, which threatens people’s safety and costs a large sum of money annually [3,4,5,6,7]. In order to control or constrain the deflections and movement of clayey soils and enhance its compressibility, strength, stiffness, the resistance to water and cracking, and other engineering properties, a variety of inorganic, organic, and biological materials such as lime [8,9,10,11], Portland cement [12,13,14,15], fly ash [16,17], granulated blast furnace slag [18,19,20], cement kiln dust [21,22], rice husk ash [23,24], polyacrylamide copolymers [25,26], bioenergy coproduct [27,28], and so on, in the form of powder or liquid, was mixed with this problematic material with or without extra water before compaction. This operation is usually referred as soil stabilization. Soil stabilization is a traditional but cost-effective technique in civil engineering, and finds its prevailing applications in pavement base, subbase, or embankment, canal or reservoir lining, shallow building foundations, and stabilized rammed earth constructions among others, especially for locations where relatively high-cost materials such as gravel and crushed stone are unavailable or have a long transportation distance while the budget of the construction project is probably limited [7,29,30].

Lime, Portland cement, and fly ash are the most commonly used binding materials in soil stabilization and they all comprise of a calcium element, which can compose two main kinds of binding gels in soil stabilization: calcium silicate hydrates (CSH) and calcium aluminate hydrates (CAH), and because of which they are called calcium-based stabilizer. These calcium-based stabilizers are usually in the form of grinded powder. During soil stabilization practice, they are combined with soil and a certain amount of water to form the soil–water–binder reaction system. It is the chemical or physico-chemical reactions (or both) in the system that essentially transforms the properties of the soils.

There are many factors affecting the efficiency of soil stabilization. Terashi [31] has categorized them into four groups: (a) characteristics of soils (soil structure, clay minerals, particle size distribution, plasticity index, cation exchange capacity, pH, contents of sulphate and organic matter, and so on); (b) characteristics of binders (binder types, dosage methodology, corresponding reaction processes such as dissolution, diffusion, and precipitation, products of chemical or physico-chemical reactions, and so on); (c) mixing and compaction procedures (pulverization, mixing uniformity, initial water content (IWC), dry density, and the like); and (d) curing procedures (temperature, air moisture, curing time, and the like). 

Among all the influential factors of soil stabilization, initial water content is of first-level importance. Firstly, the reaction process of the soil–water–stabilizer reaction system can be simplified as dissolution-precipitation [32,33,34], in which calcium-based binding materials and reactive minerals of soil first dissolve into water and then precipitate on the surfaces of soil particles to fill the soil pores (macropores and micropores [35]). Therefore, without water the reaction in the soil–water–stabilizer system will not occur. Secondly, water is a strong polar molecule and has a very powerful affinity to soil minerals. Once water goes into the soil structure, a diffused double-layer (DDL) microstructure [36,37] is formed around soil particles and influences the pore size distribution, matric suction, compressibility, and shear strength of the soils.

Initial water content as the first-considered and quality-controlled factor in soil stabilization, has been studied by other researchers. Most of the research focuses on the effects of initial water content (IWC) on the strength property of the soil treated by stabilizers, but different studies report different results and conclusions. For example, Ramesh and Sivapullaiah [38] compared the development of strength of black cotton soil (a type of vertisol with high volume change capacity) stabilized by lime under different IWCs and found that the strength of lime-stabilized black cotton soil increases rapidly when the specimens are compacted at a water content slightly lower than the optimum moisture content (the water content corresponding to the maximum dry density on the curve of Proctor compaction test). However, Guo et al. [39] analyzed the influence of IWC on the strength of lime-modified expansive soil, and considered that the optimum IWC in the construction of lime-modified expansive soil should be about 3 percentage points higher than the optimum water content. Consoli et al. [40] studied the effects of IWC on the strength of a lime-treated sandy lean clay and drew the conclusion that at the same curing age, the strength was not affected by the IWC. Besides, Consoli et al. [41] also investigated the effects of IWC on the strength of cement-stabilized clayey sand and found that the unconfined compressive strength (UCS) first increased, then was followed by a decrease with the increase of IWC. He speculated that this was attributed to the different structure formed during compaction where IWC played a fundamental role. Arora and Aydilek [42] investigated the relation between UCS and IWC of a cement-treated sandy soil-fly ash mixture and concluded that after the same curing period, the increase of IWC generally resulted in the decrease of the UCS. In addition, they ascribed this to the cementitious reactions, in which higher water/cement ratio impaired the strength development. As a result, these contradictory results could not lead to a defined recognition on the determination of optimum IWC in soil stabilization and there was not enough information in the existing research to explain the reason why the IWC affects the strength evolution of the stabilized soils.

Herein, the objective of this study is to investigate the effects of IWC on the mechanical properties (compressibility and strength), microstructure, and composition of a lean clay soil stabilized by a compound calcium-based stabilizer comprised of cement, lime, and fly ash, and explain how the microstructure and composition variation of the soil-stabilizer mixture with different IWCs affects the mechanical properties. To achieve this objective, a series of static compaction tests, unconfined compression tests, optical microscope observations, Environment Scanning Electron Microscope (ESEM) scanning, Environment Scanning Electron Microscope combined with Energy Dispersive X-ray (ESEM-EDAX) analysis, and X-ray Diffraction (XRD) were conducted. Hopefully, the results of this manuscript can help in the determination of optimum initial water content of soil stabilization practice.

## 2. Materials and Methods

### 2.1. Material Properties

#### 2.1.1. Soil

**Figure 1:**
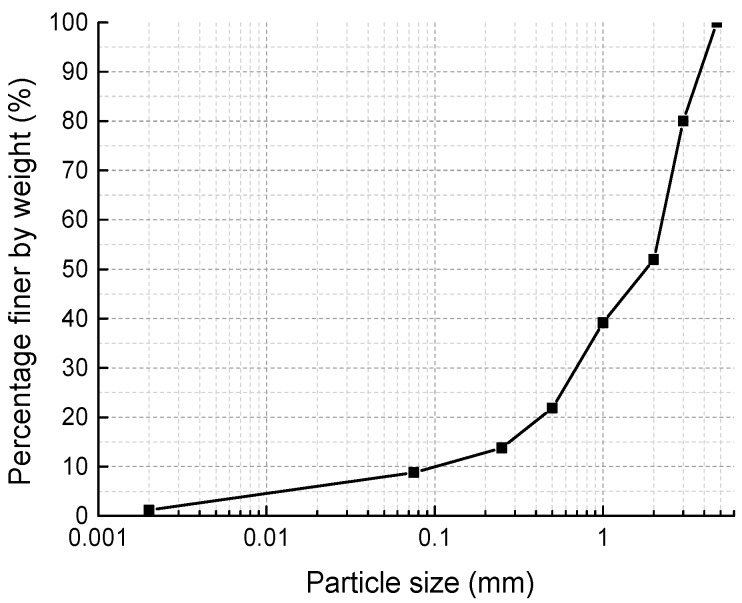
Soil particle size distribution.

**Figure 2:**
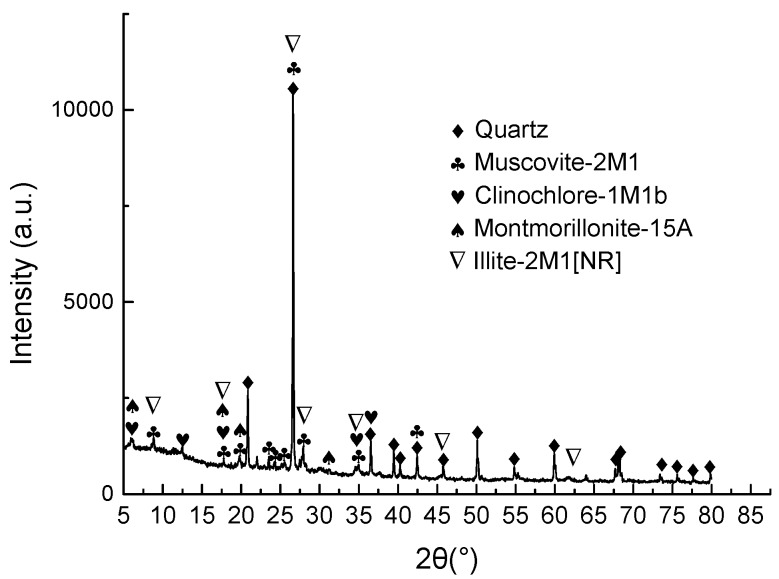
Principal mineral composition of soil.

The soil used in the tests was a natural yellowish-brown muddy soft soil taken (according to ASTM D6282/D6282M-14 [43]) from the bottom of a 1.5 m deep borrow pit near Hangzhou, China, which did not contain large particles of sand, gravel, or organic matter. After drying in the oven at the temperature of 105 °C for 3 days, the soil was pulverized to pass No. 4 (4.75 mm) sieve and deposited in a sealed plastic drum for the tests according to JTG E51-2009 [44]. The particle size distribution of the dried soil is shown in Figure 1. Table 1 shows the physical properties of the soil. Combining XRD with ESEM-EDAX analysis, it can be found that the soil mainly contained clay minerals of clinochlore, montmorillonite, and illite; and other minerals such as quartz and muscovite. The principal mineral composition of the soil is marked on the XRD pattern shown in Figure 2. The organic content in the soil was low. According to the Unified Soil Classification System (ASTM D2487-2017 [45]), the soil is classified as lean clay (CL) type.

#### 2.1.2. Compound Calcium-Based Stabilizer

**Figure 3:**
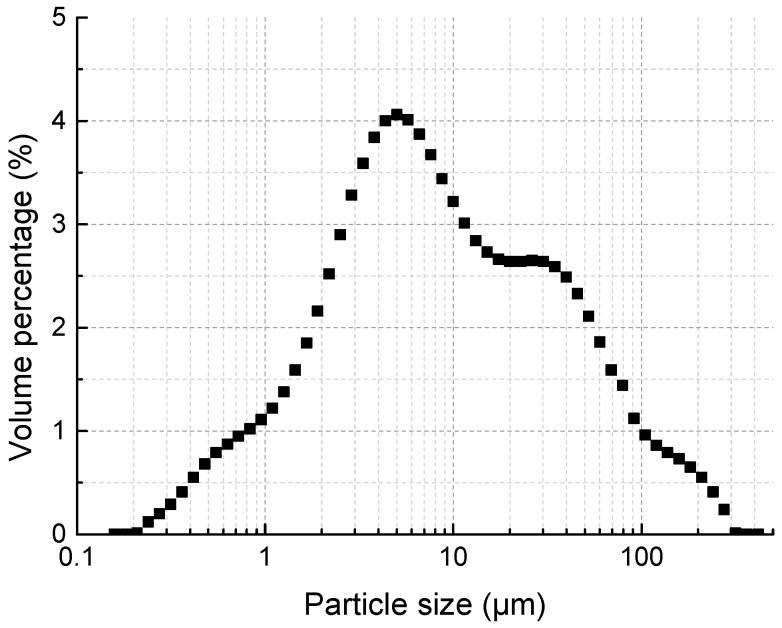
Particle size distribution of compound calcium-based stabilizer.

The calcium-based stabilizer used in the study was composed of cement, lime, and fly ash. The cement was Type 325 Ordinary Portland cement, which was produced by local Qianchao Portland Cement Company (Hangzhou, China). The lime used in the research was provided by Hangzhou Tuohai Corporation (Hangzhou, China), which was a finely ground lime powder with 85% total content of CaO and MgO. The fly ash was high calcium Class C fly ash obtained from Shaoxing Shangyu Hangzhou-union cogeneration Co., Ltd. (Shaoxing, China). Table 2 presents the chemical composition of the three additives. The particle size distribution of the mixed calcium-based stabilizer is shown in Figure 3.

### 2.2. Experimental Program

The experimental program included two parts. First, the effects of IWC on the compaction properties and UCS of the soil calcium-based stabilizer mixture were investigated by static compaction tests and unconfined compression tests. Then, the variation of microstructure and composition of the mixture with different IWCs were studied by optical microscope observation, ESEM scanning, ESEM-EDAX analysis, and XRD spectra analysis.

#### 2.2.1. Mixture Design

**Figure 4:**
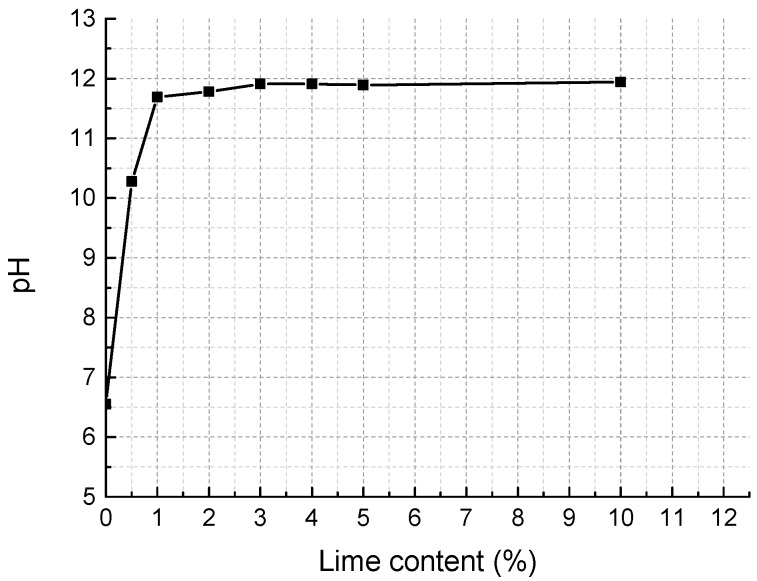
pH variation with lime content.

**Figure 5:**
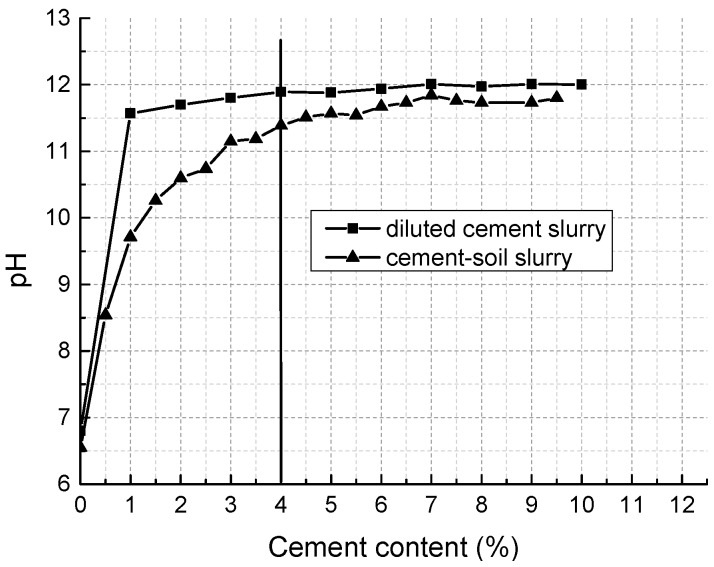
pH variation with cement content.

As is shown in Table 1, the soil is slightly acidic with a pH of 6.55. According to former studies [46,47], cement cannot fully hydrate and harden to form CSH and CAH gels until the pH arrives at a certain value. Therefore, we conducted a series of pH tests using the method presented by Eades and Grim [48] to determine reasonable initial lime and cement content. Figure 4 shows that lime at the weight of 1% dry soil can promote the pH of soil slurry up to 12.3 and after that, the increase of lime content cannot change pH significantly, so the initial lime content was set to 1% weight of dry soil. Figure 5 indicates that in the cement content range studied, when cement content reaches 4%, the pH value of diluted cement slurry is stable and the cement-soil slurry curve is not far from that of cement-soil slurry. Thus, 4% weight of dry soil was chosen to be the initial cement content. The content of fly ash, which aims to provide pozzolans, was set to be 1% weight of dry weight. Considering that fly ash can consume a certain amount of lime by pozzolanic reaction, the lime content was accordingly adjusted to 2% weight of dry soil. Consequently, the mixture was designed to be cement:lime:fly ash: dry soil equal to 4:2:1:100. Afterwards, we conducted a series of unconfined compressive tests with four different combinations of cement, lime, fly ash, and dry soil (4:1:1:100; 4:2:1:100; 3:3:1:100; 2:4:1:100). The results show that the combination with cement: lime: fly ash: dry soil equal to 4:2:1:100 has the highest unconfined compressive strength, and was thereby adopted as the final mixture proportion for the tests.

#### 2.2.2. Molding Points Design

**Figure 6:**
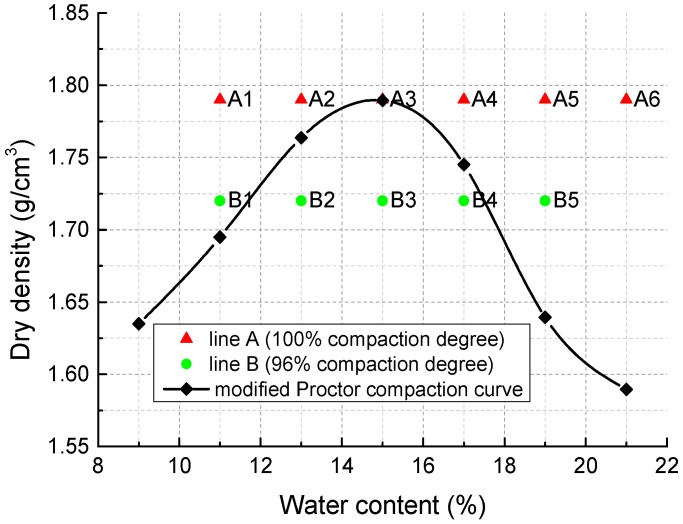
Modified Proctor compaction curve and the molding points.

As the reference point, the maximum dry density and optimum moisture content of the mixture were tested to be 1.79 g/cm^3^ and 15%, respectively, by modified Proctor compaction test (Figure 6). Specimens with different IWCs (11%, 13%, 15%, 17%, 19%, 21%) and two dry densities, 1.79 g/cm^3^ (100% compaction degree) and 1.72 g/cm^3^ (96% compaction degree), were made for the tests. The molding points (line A and B) are shown in Figure 6. For each dry density and water content, there are 30 parallel specimens.

#### 2.2.3. Specimen Molding Procedures

The specimens made in the study were cylindrical, 50 mm high, and 50 mm in diameter. During the molding process, each specimen was strictly made according to the following molding procedures:

- First, the water contents of the oven-dried soil and the calcium-based stabilizer were measured with the method of T0801-2009 in standard [44].
- Then, the amount of dry soil and stabilizer needed for 6 specimens (controlled by the blender used) was calculated, weighed, and mixed by the blender for about 5 min until the dry mixture was uniformly consistent.
- Next, the calculated volume of water was added and mixed for another 5 min until a homogenous soil-stabilizer mixture formed. After that, the mixture was carefully covered with a sealing film to prevent moisture loss.
- Finally, the quantity of the mixture for one specimen was weighed and put in the mold that had two cylindrical compaction blocks.

#### 2.2.4. Static Compaction Test

The compaction test was performed on a 30 kN hydraulic pressing machine according to JTG E51-2009 [44]. After being filled up, the mold was rapidly moved on the pressing machine to be statically compacted. The compaction process was displacement-controlled with a displacement rate of 1 mm/min. During the compaction process, the compaction data of all specimens were recorded in detail by the sensors (sampling frequency of 50 Hz) installed on the pressing machine. When the specimens were compacted to the designated dimension of 50 mm high and 50 mm in diameter, the load was kept stable for two minutes before unloading. Then, the specimens were demolded and the mass, height, and diameter of each specimen was measured and documented.

#### 2.2.5. Specimen Curing Process

To make comparisons, after compaction half of the specimens were placed in plastic sealing bags to avoid external moisture intrusion and the other half were not treated. Then, all the specimens were transferred to the curing room with a temperature at 20 ± 1 °C and humidity at 95 ± 5% in accordance with JTG E51-2009 [44]. Each of the 6 specimens (3 in bags and the other 3 without bags) were marked as a group. Thus, specimens with the same IWC and dry density were divided into five groups which were respectively cured to 1, 3, 7, 14, and 28 days. In order to only investigate the effects of IWC, all the specimens were kept as they were after different curing ages and they were not submerged into water to saturate.

#### 2.2.6. Unconfined Compression Test

The unconfined compression tests were conducted on the 30 kN hydraulic pressing machine at a displacement rate of 1 mm/min according to ASTM D2166 [49]. Before the unconfined compression test, the mass, height, and diameter of each specimen were measured again.

### 2.3. Microstructure and Composition Research

After the compression test, all the tested specimens were submerged into anhydrous ethyl alcohol for 1 day and oven-dried at 70 °C for another day to terminate the hydration process. Small parts from the unbroken district of the specimens were carefully taken out for microstructure and composition research.

#### 2.3.1. Optical Microscope Observation

To quickly and simply investigate the structure change of the dried soil, calcium-based stabilizer, and soil-stabilizer mixture with the variation of IWC in real time, the optical microscope observation was performed. The optical microscope used in the study is the Lecia DM750 (Lecia Microsystems Inc., Buffalo Grove, IL, America) equipped with the Leica ICC50 (Lecia Microsystems Inc., Buffalo Grove, IL, America) digital camera module, which can readily take photos and preserve the images. During observation, the soil, stabilizer, and the mixture were evenly placed on an object slide as a thin layer and there was not a coverslip on them. To protect the microscope, the object lens cannot be pushed too close and thus the magnification times was limited to 40.

#### 2.3.2. ESEM Scanning and ESEM-EDAX Analysis

The morphological structure of the specimens cured in plastic bags, in a much smaller scale than that of the optical microscope, was obtained by ESEM scanning. The ESEM used here was FEI Quanta 650 FEG (FEI., Hillsboro, OR, America) with an acceleration voltage range of 200 V–30 kV and maximum beam current of 200 nA. The ESEM was also equipped with an EDAX detector, by which the element composition of the designated points of the specimens was acquired.

#### 2.3.3. XRD Analysis

The mineral change of the specimens with different IWCs at different curing ages was analyzed by means of powder XRD. The XRD patterns were collected by PANalytical X’Pert PRO diffractometer (PANalytical B.V., Almelo, The Netherlands) with Cu-Kα radiation (λ = 1.5418 Å); exploration range 5° to 80°·2θ; steps of 0.026°·2θ; and goniometer speed of 0.001°·2θ·s^−1^.

## 3. Results

### 3.1. Static Compaction Test Results

**Figure 7:**
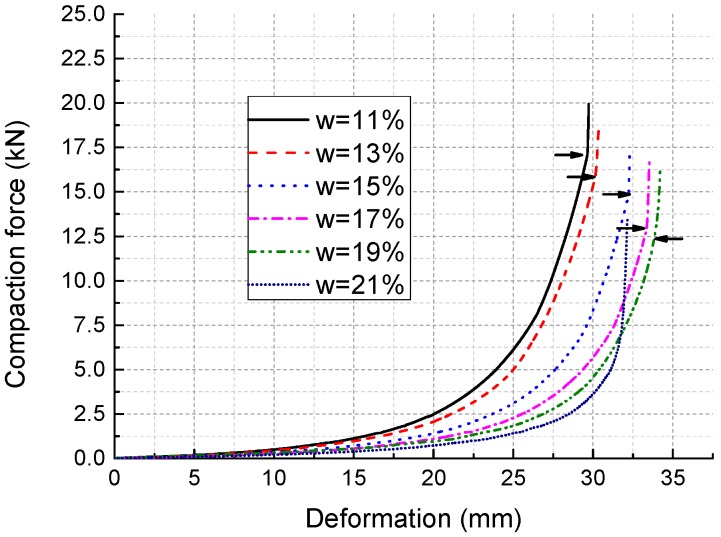
Typical compaction curves.

**Figure 8:**
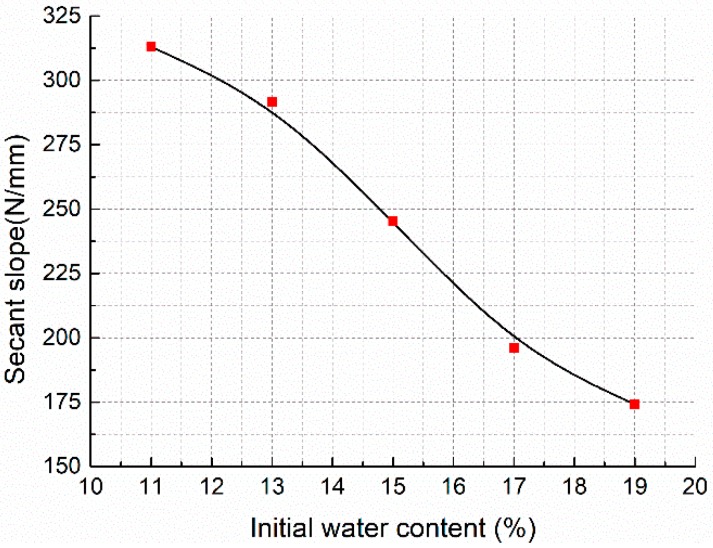
The effect of initial water content (IWC) on secant slope.

Although there are some differences among each curve even in the same IWC group, typical compaction curves of specimens with different IWCs are shown in Figure 7. The small horizontal arrows on the curves mark the points where the specimens are compacted to the designated dimension. The curve for IWC at 21% does not have an arrow because the specimens were saturated during compaction and could not be fully compacted. Figure 8 shows that as the IWC increases from 11% to 19%, the maximum compaction force decreases from around 17 to 12 kN, while the total axial deformation increases from about 29 to 34 mm. The slope of each curve, which reflects the soil stiffness under different stress states, also varies slightly with different IWCs. Figure 8 shows the secant slopes from original point to points of half maximum compaction force. It indicates that the mean stiffness of the soil decreases in the form of cubic function as IWC increases from 11% to 19%.

**Figure 9:**
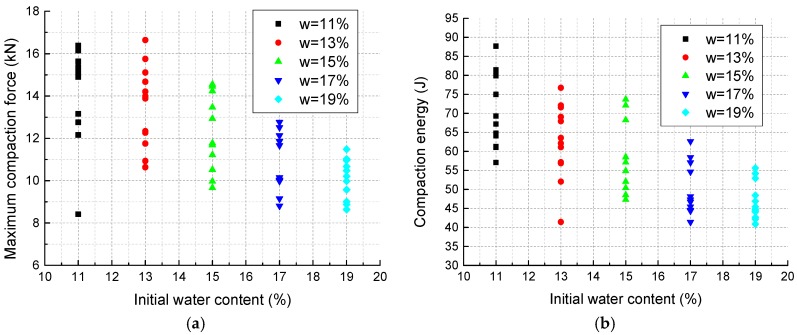
Results of static compaction test: (**a**) maximum compaction force; (**b**) compaction energy.

To better understand the effects of IWCs on the compaction properties of the mixture, the maximum compaction force (Figure 9a) and compaction energy (Figure 9b) of two groups of specimens at 100% compaction degree are further analyzed. The compaction energy is computed by the integral of the compaction curve with the equation:

(1) $$E_{c}=\overset{n}{\underset{i=1}{\sum }}0.5(F_{i}+F_{i-1})(x_{i}-x_{i-1})$$

where the $E_{c}$ is the compaction energy; $F_{i}$ and $F_{i-1}$ are the number *i* and *i −* 1 compaction force, respectively; $x_{i}$. and $x_{i-1}$ are the number *i* and *i −* 1 displacement recorded by sensors.

It is observed that both the maximum compaction force and compaction energy decrease linearly with the increase of IWC. The largest decline percentages for the maximum compaction force and compaction energy are calculated to be 45.9% and 53.4%, respectively.

### 3.2. Unconfined Compression Test Results

**Figure 10:**
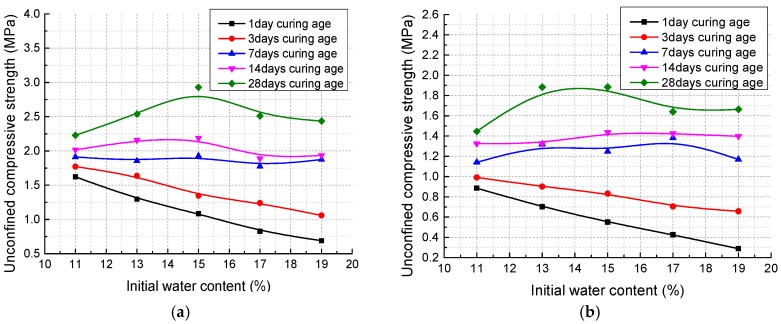
Effects of initial water content on unconfined compressive strength of specimens cured in plastic bags: (**a**) dry density 1.79 g/cm^3^; (**b**) dry density 1.72 g/cm^3^.

Figure 10 presents the relationship between UCS and IWC at different curing ages (1day, 3days, 7 days, 14 days, and 28 days) under two curing conditions (with and without external water) and two degrees of compaction (100% and 96%). The test results for specimens cured without external water supply are shown in Figure 10a (100% compaction degree) and Figure 10b (96% compaction degree). Both of the two figures show the same variation trend of UCS regardless of the compaction degree:

1. At early curing age (1 day and 3 days) the UCS decreases linearly as IWC increases from 11% to 19%;
2. After curing for 7 days and 14 days, the UCS value does not change significantly with the variation of IWC;
3. Through a curing time of 28 days, a parabolic relationship between UCS and IWC can be observed: the UCS first increases and then decreases with the increase of IWC, reaching peak value at 15% IWC, which is equal to the optimum water content derived from the modified Proctor compaction test.

Additionally, it can be found that in the first 7 days the UCS develops fast and the increase rate of UCS accelerates with higher IWC; but afterwards the strength development gets slow and the increase rate first enlarges then reduces in the variation range of IWC, reaching maximum at 15% IWC.

**Figure 11:**
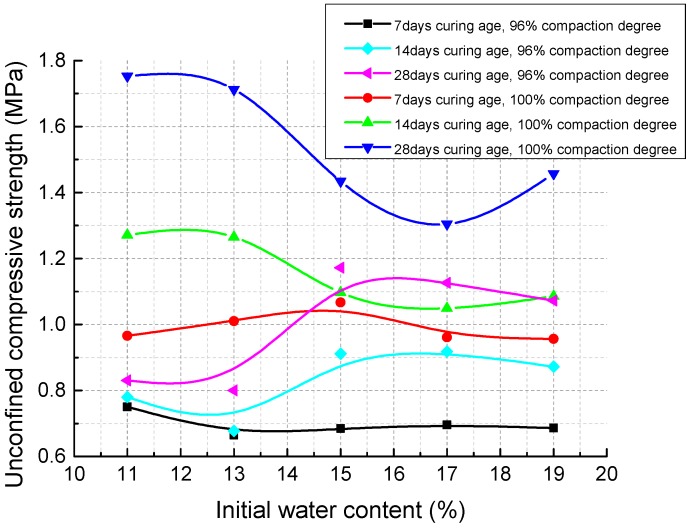
Effects of initial water content on unconfined compressive strength of specimens cured in moist air.

As a comparison, Figure 11 presents the unconfined compression test results for specimens cured in moist air. It can be clearly found that after a curing time of 14 and 28 days, for specimens with 96% degree of compaction, the highest UCS value is obtained when the IWC is 15%, which equals the optimum water content of modified Proctor compaction test; whereas under 100% degree of compaction, the specimens compacted at lower IWCs (11% and 13%) tend to have higher UCSs.

### 3.3. Optical Microscope Observations

**Figure 12:**
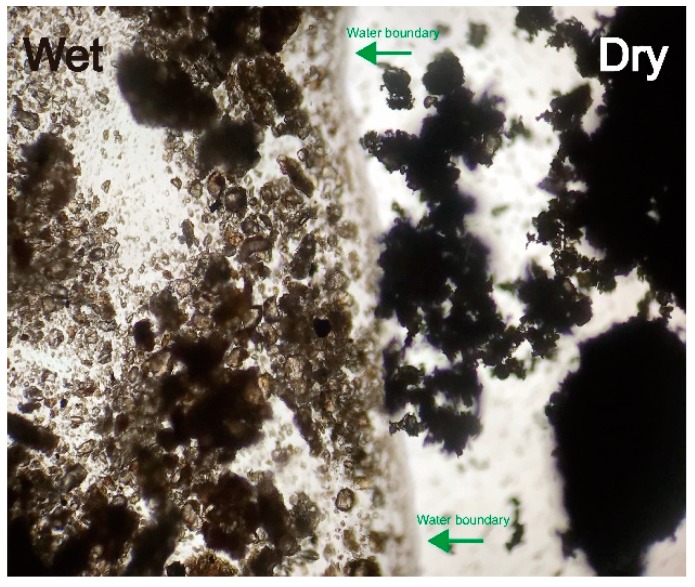
Structure of wet soil (**left**) and dry soil (**right**).

Figure 12 shows the comparison of the structure of wet soil and dry soil. When the soil is oven-dried to a water content about 3%, smaller particles and aggregates are generally attracted to the surfaces of larger ones by attractive forces (mainly electrostatic forces according to former research [1,50]), forming a spatial and porous structure. However, after water intrusion into the dry soil, the original structure immediately breaks down to a dispersed fabric with clearer particle boundaries and better transparency. When part of the wet soil is moved with the tip of toothpick, the adjacent soil particles do not move. However, when the dry soil is moved in the same way, the adjacent soil matric particles and aggregates in a certain distance move together. This optical microscope observation indicates that water can decrease the attractive force between soil particles and make the soil structure easier to change.

**Figure 13:**
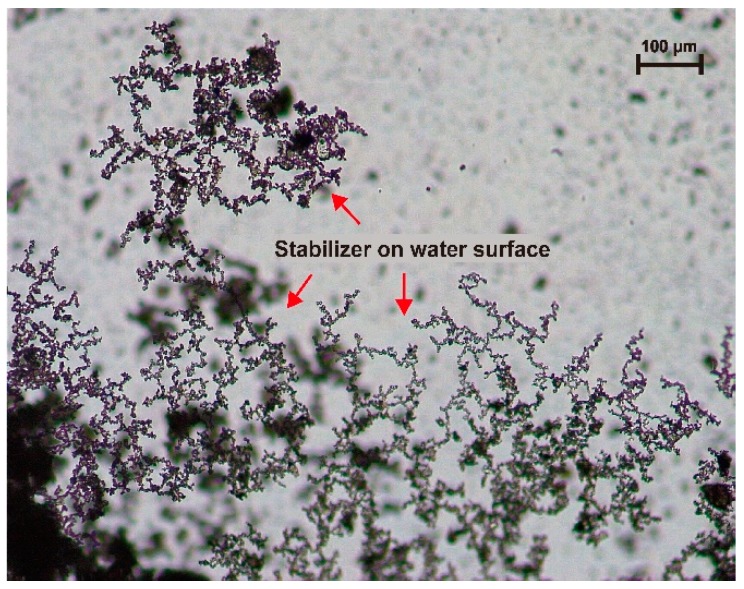
Stabilizer on the surface of water.

Another interesting phenomenon from Figure 13 is that when the water boundary gets close to the dry soil, the whole aggregate will be pulled quickly into the water, while when the water boundary is approached by the stabilizer in the same way, the stabilizer particles have difficulty in being attracted into the water and some of them are even just absorbed on the water surface. According to the theory of surface thermodynamics [51,52,53], this phenomenon indicates that the soil particles have higher surface energy than the stabilizer particles and can be more easily wetted by water.

**Figure 14:**
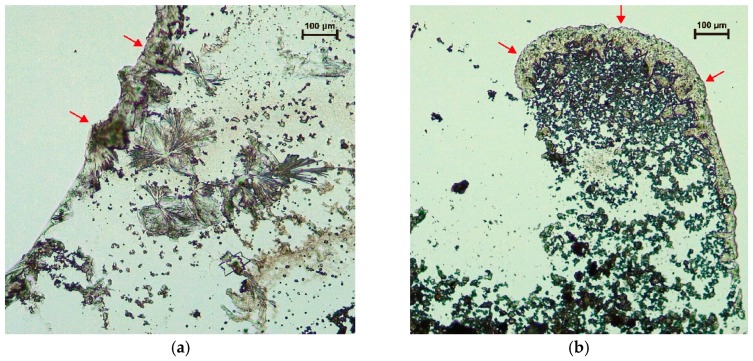
Reaction products of calcium-based stabilizer (the red arrows pointing); (**a**) reaction time of 0.5 h; (**b**) reaction time of 2 h.

Figure 14 exhibits some reaction products of the stabilizer and it seems that the reaction products tend to be principally formed on the boundary of the water, though there are a few pieces of radial formations which look like ettringite located in the water area, shown in Figure 14a.

### 3.4. ESEM Scanning and ESEM-EDAX Analysis

**Figure 15:**
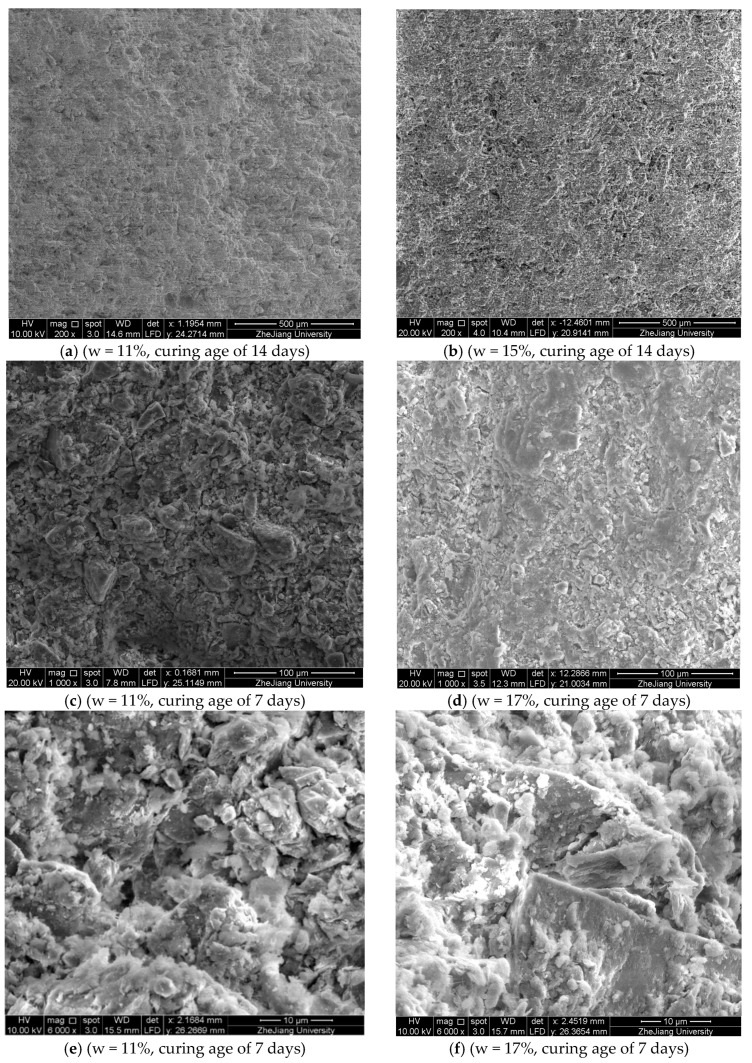
Effects of initial water content on microstructure.

The microstructure of the specimens was investigated by ESEM scanning (Figure 15). The results show that as the IWC increases, the number of large aggregates and agglomerations reduces while the quantity of individual particles increases; and in turn the number of large inter-aggregate pores decreases, leading to the soil structure becoming fine and dense (Figure 15a–d). In addition, it is observed that in 1 day- and 3 day-specimens, the hydration products are too few to be easily found. Only in specimens curing for 7 days will the hydration products of the calcium-based compound stabilizer start to notably come up (Figure 15e,f). Furthermore, owing to the hydration products mainly generating in the pores of soil matric particles, specimens with higher IWCs which have a more dispersed pore structure seem to have a much more homogeneous structure (Figure 15c–f).

**Figure 16:**
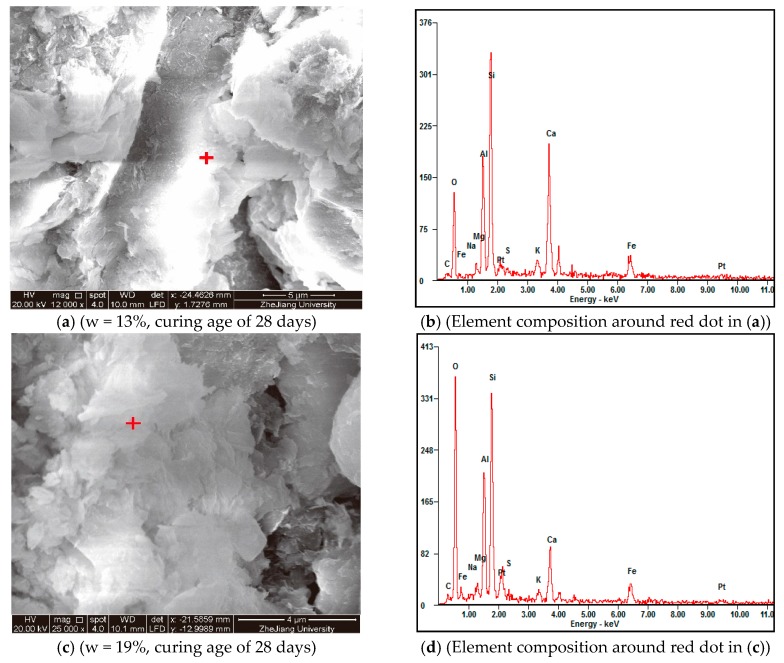
Effects of initial water content on chemical composition.

During the ESEM scanning process, we also used EDAX to identify the element composition of the hydration products in different areas of the specimens (Figure 16b,d). It was found that though the hydration products (which are already known as CSH and CAH) are mainly composed of oxygen, silicon, aluminum, and calcium, the percentage of each element in different scanning spots varies a lot even in the same specimen. However, generally, it seems that for the hydration products of specimens with higher IWC, the percentage of oxygen element tends to be a little higher, probably because more free water transforms into structural water during the hydration process.

### 3.5. XRD Results

**Figure 17:**
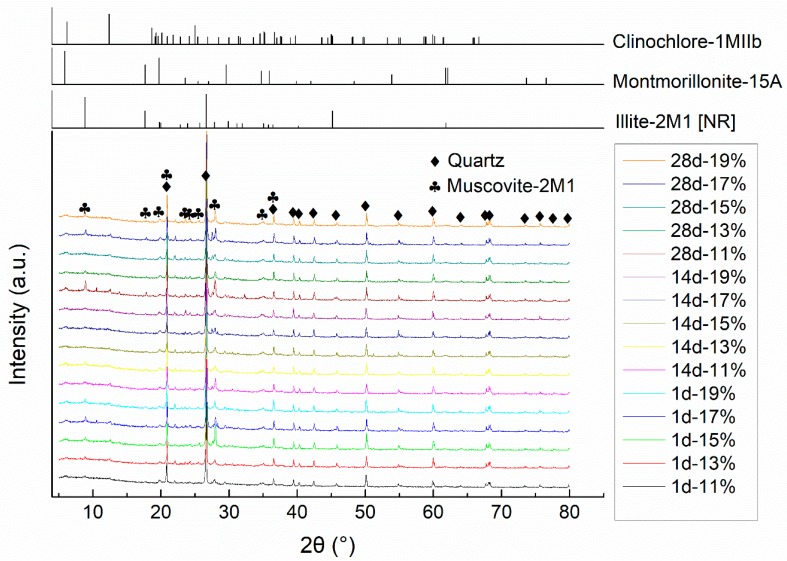
Effects of initial water content on mineral composition.

Figure 17 presents the X-ray diffractograms of powder samples taken from specimens cured to 1, 14, and 28 days in plastic bags. It can be found that the diffractograms of samples with different IWCs and various curing ages exhibit little difference with each other, especially for the characteristic peaks of primary minerals (principally quartz and muscovite in the soil), which indicates that IWC has little effect on the crystallized primary mineral composition of the soil-stabilizer mixture. However, the characteristic peaks of clay minerals seem to change without a clear trend, which might be ascribed to sampling differences and inherent inhomogeneity of the natural soil. The expected dispersed characteristic peaks of poorly crystallized hydration products, CSH and CAH [54], are not notable, which may be attributed to the relatively small addition of calcium-based compound stabilizer and insufficient crystallization time.

## 4. Discussion

Combining the mechanical property study with the microstructure and composition research, the effects of initial water content on the soil-stabilizer mixture studied can be revealed as follows.

### 4.1. The Effects of Initial Water Content on the Compaction Process

The results of optical microscope observation identify the foundational effect of the molding water: it breaks down the soil aggregates and agglomerations into individual particles and makes the soil structure more easily able to be changed. This is also confirmed by other research [55,56,57] and can be explained by the diffused double layer (DDL) theory. More water enlarges the thickness of the DDL of the soil particles and even free water will come up, lessening the shear resistance of the soil. As a result, on the scale level of the whole mixture, the stiffness of the mixture declines (Figure 8) and if there is no excess pore water pressure, it will need less force and energy to densify the mixture, which is testified by the static compaction test (Figure 9). Meanwhile, more water in the mixture means more individual soil particles exist and thus during the densification process, small particles can more easily move to the pores between larger particles or aggregates to minify the pores, which was identified by the ESEM scanning (Figure 15) and other relevant research [57,58,59,60]. Therefore, right after compaction, the specimens with different IWCs have different original structures: specimens with higher initial water content have less aggregates and agglomerations, and a smaller proportion of large pores.

### 4.2. The Effects of Initial Water Content on Strength Development

During the curing process, the water reacts with the calcium-based stabilizer to produce the bonding gels. Specimens with different IWCs have different reaction rates (Figure 10 and Figure 11). The produced gels change the mixture structure to yield different a UCS at different curing ages for different specimens. The UCS is just the reflection of the structural properties of the mixture at a certain time.

For the specimens cured without external water, in the earlier days (1 day and 3 days) of the curing process, the stabilizer has not fully reacted and the mixture structure mainly maintained what they were after compaction, which is confirmed by the ESEM scanning. Thus, the strength of the mixture is principally dependent on the strength of the soil structure, which is controlled by the average thickness of the water membrane (DDL and free water). As initial water content increases, the average water membrane is thicker. Consequently, the UCS decreases linearly with the increase of IWC, as shown in Figure 10 and Figure 11. However, as water content increases, there is a bigger probability to form continuous water passage, which facilitates the cation exchange, dissolution, and diffusion process of the soil–water–stabilizer reaction system and thus, the increase rate of UCS in the first 7 days is higher for the specimens with higher IWC. The UCS value does not change significantly with the variation of IWC at the curing age of 7 days. After that, as the pores are gradually filled by reaction products and free water is lacking, the increase rate for all the specimens decreases.

At 28 days curing age, for specimens with lower IWC, there are more large pores and some of them cannot be fully filled and bonded (Figure 15a,c); while in the specimens with higher IWC, though a more homogeneous structure is observed (Figure 15b,d), higher IWC excessively destroys the soil structure and more free water remains in the water membrane of the soil particles. This may be why the UCS first increases and then decreases with the increase of IWC at 28 days curing age.

For the specimens cured in moist air, the water was continuously supplied by an external water source until the specimens were saturated. Therefore, the inherent aggregates and agglomerations of the soil can be totally wetted during the curing process and the strength contributions of the soil structure are weakened. As a result, at the same compaction degree, the UCS of specimens cured in moist air significantly reduced compared to that of specimens cured without external water. Under different compaction degrees at the same curing age, the UCS variation with IWC is different (Figure 11) and this may be attributed to the different original structures after compaction. It can be speculated that there must be an optimum original mixture structure corresponding to certain initial water content under different compaction degrees that is beneficial to the strength development, which needs further investigation.

From the discussion above, it can be concluded that the determination of IWC in soil stabilization practice should consider three issues: compaction, curing environment, and the targeted value of specific engineering properties; rather than only compaction. It is recommended that when a soil is stabilized by a stabilizer that can react with water, for a given dry density and a given curing environment, a series of IWCs should be tested to find the appropriate compaction energy and the optimum IWC.

## 5. Conclusions

Initial water content is an important factor affecting engineering properties such as strength, stiffness, and compressibility of the soil stabilized by the calcium-based stabilizer in the research. The following conclusions can be drawn from the study:

- In the range of initial water content studied, both the compaction energy and the maximum compaction force decrease linearly with the increase of initial water content, and the largest reduction is 53.4% and 45.9%, respectively.
- As the initial water content increases from 11% to 19%, there are less soil aggregates or agglomerations, and a smaller proportion of large pores in the mixture structure after compaction.
- For specimens cured without external water supply, regardless of the compaction degree, after a curing age of 28 days, the highest unconfined compressive strengths were acquired at initial water content of 15%, which is equal to the optimum water content derived from the modified Proctor compaction test. Higher initial water content enlarges the increase rate of the unconfined compressive strength in the first 7 days; after that, the increase rate first increases then decreases, and acquires maximum at initial water content of 15%.
- For specimens cured in moist air, the optimum initial water contents are 11% under 100% compaction degree, and 15% under 96% compaction degree, respectively.
- As the initial water content increases, the percentage of the oxygen element tends to increase in the reaction products of the calcium-based stabilizer.
- In the curing ages studied, the initial water content did not notably change the primary mineral composition of the soil-stabilizer mixture.

## Figures and Tables

**Table 1:** Physical properties of soil sample.

Natural Dry Density (g/cm^3^)	Dried Moisture Content (%)	Specific Gravity	Liquid Limit (%)	Plastic Limit (%)	Plasticity Index (%)	Activity of Clay	pH
1.64	2.94	2.69	37.8	19.3	18.5	2.02	6.55

**Table 2:** Chemical composition of the calcium-based stabilizers.

Materials	Chemical Compositions (Mass Fraction, %)								
	SiO_2_	Al_2_O_3_	Fe_2_O_3_	CaO	Na_2_O	K_2_O	MgO	TiO_2_	SO_3_
Portland cement	18.04	8.79	4.96	54.14	0.12	0.32	3.56	-	1.77
Lime	-	-	-	86.26	-	-	0.68	-	-
Fly ash	11.61	21.73	1.75	40.28	0.95	1.36	0.49	1.66	0.61
